# Interplay among differential exposure to *Mycobacterium leprae* and TLR4 polymorphism impacts the immune response in household contacts of leprosy patients

**DOI:** 10.3389/fimmu.2023.1130137

**Published:** 2023-04-28

**Authors:** Eloisa Helena Medeiros Cunha, Pedro Henrique Ferreira Marçal, Rafael Silva Gama, Lorena Bruna Pereira de Oliveira, Roberta Olmo Pinheiro, Euzenir Nunes Sarno, Joaquim Pedro Brito-de-Sousa, Márcio Luís Moreira de Souza, Jessica Kathleen Fairley, Thaisa Netto Souza Valente, Cibele Velloso-Rodrigues, Olindo Assis Martins-Filho, Dirce Ribeiro de Oliveira, Lucia Alves de Oliveira Fraga

**Affiliations:** ^1^ Universidade Federal de Juiz de Fora, Governador Valadares, MG, Brazil; ^2^ Universidade Vale do Rio Doce – Univale, Department of Health Sciences, Governador Valadares, MG, Brazil; ^3^ Division of Infectious Diseases, Department of Medicine, School of Medicine, Emory University, Atlanta, GA, United States; ^4^ Laboratório de Hanseníase, Instituto Oswaldo Cruz, Fundação Oswaldo Cruz –FIOCRUZ-RJ, Rio de Janeiro, RJ, Brazil; ^5^ Grupo Integrado de Pesquisas em Biomarcadores, Instituto René Rachou, FIOCRUZ-Minas, Belo Horizonte, MG, Brazil

**Keywords:** leprosy, chemokines and cytokines, immunological factors, genetic factors, *TLR4* gene

## Abstract

**Introduction:**

The aim of the present study was to investigate the association between the single nucleotide polymorphism (SNP) rs1927914 A/G in *TLR4* gene and the immunological profile of household contacts (HHC) of leprosy patients. Leprosy classification is usually complex and requires the assessment of several clinical and laboratorial features.

**Methods:**

Herein, we have applied distinct models of descriptive analysis to explore qualitative/quantitative changes in chemokine and cytokine production in HHC further categorized according to operational classification [HHC(PB) and HHC(MB)] and according to *TLR4*SNP.

**Results and discussion:**

Our results showed that *M. leprae* stimuli induced an outstanding production of chemokines (CXCL8;CCL2; CXCL9; CXCL10) by HHC(PB), while increase levels of pro-inflammatory cytokines (IL-6; TNF; IFN-γ; IL-17) were observed for HHC(MB). Moreover, the analysis of chemokine and cytokine signatures demonstrated that A allele was associated with a prominent soluble mediator secretion (CXCL8; CXCL9; IL-6; TNF; IFN-γ). Data analysis according to *TLR4* SNP genotypes further demonstrated that AA and AG were associated with a more prominent secretion of soluble mediators as compared to GG, supporting the clustering of AA and AG genotypes into dominant genetic model. CXCL8, IL-6, TNF and IL-17 displayed distinct profiles in HHC(PB) *vs* HHC(MB) or AA+AG *vs* GG genotype. In general, chemokine/cytokine networks analysis showed an overall profile of AA+GA-selective (CXCL9–CXCL10) and GG-selective (CXCL10–IL-6) axis regardless of the operational classification. However, mirrored inverted CCL2–IL-10 axis and a (IFN-γ–IL-2)-selective axis were identified in HHC(MB). CXCL8 presented outstanding performance to classify AA+AG from GG genotypes and HHC(PB) from HHC(MB). TNF and IL-17 presented elevated accuracy to classify AA+AG from GG genotypes and HHC(PB) (low levels) from HHC(MB) (high levels), respectively. Our results highlighted that both factors: i) differential exposure to *M. leprae* and ii) *TLR4* rs1927914 genetic background impact the immune response of HHC. Our main results reinforce the relevance of integrated studies of immunological and genetic biomarkers that may have implications to improve the classification and monitoring of HHC in future studies.

## Introduction

Leprosy is a chronic granulomatous disease caused by *Mycobacterium leprae* infection that affects the skin, peripheral nerves and nasal mucosa presenting a wide range of clinical manifestations (1). Considering the broad spectrum of clinical manifestations, leprosy classification is usually complex and requires the assessment of clinical, histopathological, microbiological, and immunological features as originally proposed by Ridley and Jopling (2). Classically, distinct patterns of immune response have been associated with dichotomous clinical manifestations (3). While patients with few skin lesions and low/absent bacillary index displayed a preserved cellular immune response, mediated by a strong IFN-γ production, patients multiple skin lesions and high bacillary load exhibited an impaired or absent cellular responses along with enhanced antibody (4). In the late 1990s, the World Health Organization (WHO) proposed a simplified operational classification system, considering the number of cutaneous lesions. According to this operational classification system, patients presenting up to five skin lesions are classified as paucibacillary (PB) while patients with more than five lesions are considered multibacillary (MB) (5).

The accurate classification of PB and MB leprosy patients is relevant to support the implementation of proper multidrug therapy and plays a key role for the success of leprosy control programs. Currently, the diagnosis of leprosy is achieved essentially by clinical evaluation. Although skin smears and histopathological tests can contribute to leprosy diagnosis, these complementary methods are usually available only in specialized reference laboratories (6). In this sense, the use of integrated platforms and algorithms to increase the sensitivity and specificity of leprosy patient classification is considered a relevant strategy to improve the leprosy diagnosis (7, 8).

Previous studies from our group have demonstrated the applicability of using immunological and genetic biomarkers associated with artificial intelligence approaches to help the classification leprosy patients as well as to monitor their household contacts (9–16). As the household contacts of MB leprosy patients presented 5 to 10 times greater risk of developing the disease as compared to the general population, a persistent and continuous follow-up of household contacts represent a relevant strategy to achieve an early diagnosis of subclinical infections (17, 18). In order to define an accurate diagnosis of subclinical infection, the introduction of innovative methodologies is useful to identify novel biomarkers with putative clinical applications. In this context, an algorithm design for a cytokine release assay of antigen-specific *in vitro* stimuli of circulating leukocytes has been proposed to classify leprosy patients and household contacts, demonstrating that immunological methods are promising tools to complement the clinical diagnosis of subclinical leprosy and ultimately contributing to effective control of the disease (16). Moreover, the use of molecular methods applied to identify single nucleotide polymorphism (SNP) of Toll-like receptors (TLRs), particularly TLR1, TLR2 and TLR4, have been reported to be associated with distinct leprosy clinical manifestations (19–23. The relationship between TLRs SNPs and the susceptibility to leprosy as well as the immunological profile of leprosy patients has been already investigated. It has been shown that while TLR2 SNP was associated with increased risk for leprosy, TLR1 and TLR4 SNP were associated with differential production with chemokine and cytokines (24).

The present study intended to investigate the association between TLR4 SNP and the immunological profile of household contacts (HHC) of leprosy patients. For this purpose, chemokines and cytokines were measured in the supernatant of *in vitro* cultured peripheral blood mononuclear cells (PBMC) from HHC categorized based on operational classification (PB and MB) and the qualitative and/or quantitative changes in their levels evaluated according to *TLR4* rs1927914 polymorphism.

## Patients, material and methods

### Study population

This is a cross-sectional investigation carried out in Governador Valadares, Minas Gerais State, Brazil, a hyperendemic area for leprosy (1.9 cases/10,000 inhabitants) (5). A total of 37 household contacts of leprosy patients (26 females and 11 males), age ranging from 5 to 74 years old were included as a convenience sampling using a non-probabilistic approach. The household contacts of leprosy patients were further categorized into two subgroups, based on operational classification according to World Health Organization, as follows: household contacts of paucibacillary patients [HHC(PB), n=10, 8 females and 2 males, age ranging from 7 to 61 years old] and multibacillary patients [HHC(MB), n=27, 18 females and 9 males, age ranging from 5 to 74 years old]. The **Table 1** provide major sociodemographic features and clinical laboratorial records of the study population.

**Table 1:** Sociodemographic features and clinical-laboratorial records of the study population.

Parameters	HHC(PB) (n=10)	HHC(MB) (n=27)
Sociodemographic Features		
Age* median (min-max)	32 (7-61)	33 (5-74)
Sex (%)		
**Males**	20	33
**Females**	80	67
Ethnicity (%)		
**White**	40	67
**Mixed**	10	21
**Black**	50	12
Neighborhood (%)		
**Urban**	40	88
**Rural**	60	22
Number of Residents (%)		
**1-2**	10	19
**3-4**	80	33
**>5**	10	48
Scholarly (%)		
**Illiterate**	20	8
**Elementary school**	70	70
**High school**	10	18
**University education**	0	4
Income^#^		
**1**	20	41
**2**	80	41
**3**	0	18
Kinship (%)		
**Yes**	50	85
**No**	50	15
Clinical-Laboratorial Records		
BCG Vaccination		
**Yes**	70	85
**No**	30	15
**WBC (cells/mm^3^)**	7,200	7,500
**Neu (%)**	62	61
**Mon (%)**	3	5
**Lym (%)**	28	31
**RBC (x10^6^/mm^3^)**	4.6	4.4
**Hemoglobin (g/dL)**	13.5	12.9
**Hematocrit (%)**	43	40
**Platelet (count x10^3^/mm^3^)**	241	247
**Vitamin D [25(OH)D] (ng/mL)**	33	29

HHC(PB), household contacts of paucibacillary leprosy patients; HHC(MB), household contacts of multibacillary leprosy patients; BCG, (Bacillus Calmette-Guerin; WBC, White blood cells; Neu, neutrophils; Mon, Monocytes; Lym, lymphocytes; RBC, Red blood cells; *Age is expressed in years old. ^#^Income is expressed in number of minimal salary equivalent to US$ 250. Laboratorial records as expressed as median values.

The inclusion of participants was based on convenience sampling using a non-probabilistic approach. There was no pattern in selecting the participants. The household contacts invited to participate in the present investigation, comprised leprosy patient companions contacted at the outpatient unit Reference Center for Endemic Diseases and Special Programs (CREDEN-PES) at the Department of Public Health of Governador Valadares municipality, MG, Brazil.

The study was submitted and approved by the Ethics Committee from Universidade Federal de Juiz de Fora – UFJF (Research Protocol CAAE #56863016.6.1001.5147). All participants have read and signed the informed consent form before the inclusion in the study.

### Whole blood sampling and PBMC culture *in vitro*

Heparinized whole blood samples (10mL) were collected from each participant and used to obtain peripheral blood mononuclear cells (PBMC) for chemokine and cytokine quantification in supernatant from *in vitro* cultures. PBMC were isolated by Ficoll Hypaque cushion gradient, washed twice and resuspended in RPMI-1640, supplemented with 10% fetal bovine serum, 2mM L-glutamine, penicillin (100U/mL) and streptomycin (100μg/mL). Cells (2x10^5^/well) were cultured in duplicates on flat-bottom 96-well plates, at 37°C, 5% of CO_2_ in humidified incubator. Parallel batches of cultures were carried out in the absence of exogenous stimuli (unstimulated culture) and in the presence of 10 irradiation-killed *M. leprae* bacilli/cell (*M. leprae*-stimulated culture). The *M. leprae* bacilli were kindly provided by the Leprosy Laboratory at FIOCRUZ-RJ and Instituto Lauro de Souza Lima - ILSL/SP, obtained as previously described by 25. Culture supernatants were harvested at 5 days for chemokine and cytokine quantification.

### Chemokine and cytokine quantification by cytometric beads array

Quantitative analysis of chemokines and cytokines was performed by Cytometric Bead Array (Human Chemokine Kit for CXCL8, CCL2, CXCL9, CCL5 and CXCL10; Human Cytokine Flex Set kit for IL-6, TNF, IFN-γ, IL-17, IL-4, IL-10 and IL-2) purchased from BD Bioscience, Pharmingen, (San Diego, CA, USA) as recommended by the manufacturer. A total of 400 events/chemokine or cytokine-specific beads were acquired on a FACSVerse*^rmTM^* Bioanalyzer (BD Bioscience, San Jose, CA, USA). Data analysis was performed using the FCAP Array multiplex assay analysis software (Soft Flow, Inc., St. Louis Park, MN, United States).

### TLR4 genotyping

The Illustra^®^ blood genomic Prep Mini Spin Kit (GE Healthcare) was used to extract DNA from the 250µL of whole blood samples from a subgroup of household contacts of leprosy patients [HHC(PB), n=10 and HHC(MB), n=23] and genotyping of SNP rs1927914 A/G in *TLR4* gene by the allelic discrimination using a validated predesigned Taqman^®^ quantitative Polymerase Chain Reaction (qPCR) from Life Technologies^®^ (Thermo Fisher, Inc). The assays were performed according to the manufacturer’s instructions and employed the 7500 Fast Real-Time PCR (Applied Biosystems^®^) system platform and the ABI software v 2.0.6 for data analysis.

### Statistical *analysis*

Conventional statistical analysis of chemokines and cytokines were carried out using the Prism GraphPad software (version 8.01, San Diego, California, USA). Student *t* or Mann-Whitney tests were employed for pairwise comparisons between HHC(PB) *vs* HHC(MB).

Additional descriptive analysis of biomarker signatures was carried out as previously reported by Marçal et al. (16), modified as follows: continuous variables expressed in pg/mL were converted into categorical data using the intrinsic median values as the cut-off ​​for unstimulated culture (CXCL8 = 6,015; CCL2 = 3,481; CXCL9 = 400; CCL5 = 822; CXCL10 = 1,214; IL-6 = 10,938; TNF = 5.3; IFN-γ = 297; IL-17 = 6.4; IL-4 = 1.0; IL-10 = 54 and IL-2 = 9.1 pg/mL) and *M. leprae*-stimulated culture (CXCL8 = 4,319; CCL2 = 3,114; CXCL9 = 324; CCL5 = 1,005; CXCL10 = 1,224; IL-6 = 10,847; TNF = 4.4; IFN-γ = 363; IL-17 = 5.5; IL-4 = 1.0; IL-10 = 55 and IL-2 = 12 pg/mL). Following, the proportion of subjects (%) with chemokine and cytokine levels above the intrinsic cut-off were calculated for each study subgroups. The chemokines and cytokines with proportion of subjects ​​above the 50^th^ percentile were underscored and considered with increased levels.

The analysis of genotype data for the SNP *TLR4* rs1927914 was carried out to check for deviation from Hardy–Weinberg equilibrium (HWE) using the online calculator, available at https://gene-calc.pl/. Comparative analysis between A *vs* G alleles was performed by Student *t* or Mann-Whitney tests. ANOVA variance analysis followed by Tukey or Kruskal-Wallis with Dunn’s post-test was used for multiple comparisons amongst HHC(PB) and HHC(MB) subgroups according to *TLR4* rs1927914 genotypes as well for dominant genetic model (AA+AG *vs* GG). In all cases, significant differences were considered at *p*< 0.05.

Assessment of integrative chemokine and cytokine networks were assembled using only significant “r” scores at p<0.05 obtained from Pearson and Spearman correlation analysis. The Cytoscape software platform (available at https://cytoscape.org) was employed to construct the circular layout networks, with nodes representing the chemokines and cytokines. Connecting edges illustrate positive (continuous lines) and negative (dashed lines) strong correlations (“r” scores ≥ |0.67|) between pairs of attributes.

The analysis of Receiver-operating Characteristic Curves (ROC) was carried out for evaluating the accuracy of chemokines and cytokine measurements to classify HHC into categories, according to operational classification and SNP *TLR4* rs1927914 polymorphism. The MedCalc software (version 18.6, Ostend, Belgium) was used for performance analysis. The Area Under the ROC Curve (AUC) was used as an indicator of global accuracy. Significance was considered at p<0.05. The cut-offs provided by the ROC curve analysis were used to assess the performance indices, including sensitivity (Se), specificity (Sp), and likelihood ratio (LR).

Exploratory heatmap constructs were assembled using conditional formatting in Microsoft Excel Office version 2016. The analysis was performed using customized functions, considering the column Row Z-score, scaled from -2.5 to +5.0. A color key (25^th^, 50^th^ and 75^th^) was used to identify clusters of subjects with distinct (low or high) levels of chemokines and cytokines in each subgroup of household contacts of leprosy patients.

## Results

### Panoramic overview of chemokines and cytokines secretion by *in vitro* cultured PBMCs from household contacts of leprosy patients

**Figure 1:**
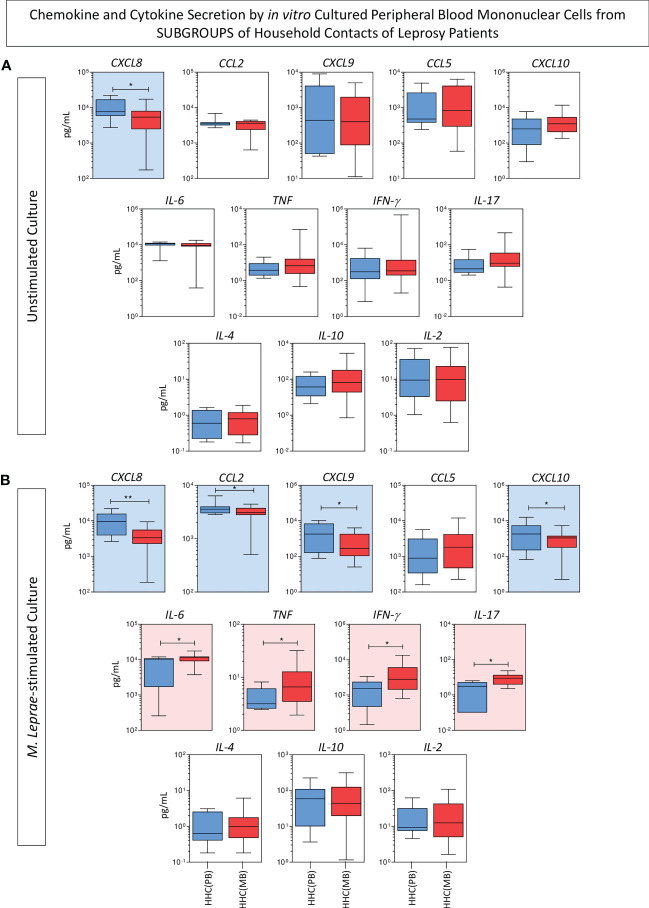
Panoramic overview of chemokines and cytokines secretion by *in vitro* cultured peripheral blood mononuclear cells from subgroups of household contacts of leprosy patients. The levels of chemokines (CXCL8, CCL2, CXCL9, CCL5, CXCL10) and cytokines (IL-6, TNF, IFN-γ, IL-17, IL-4, IL-10 and IL-2) were measured in the supernatant collected upon *in vitro* culture of PBMC from household contacts on paucibacillary [HHC(PB) = 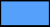, n=10] and multibacillary patients [HHC(MB) = 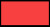, n=27]. Data were obtained **(A)** in the absence of exogenous stimuli (unstimulated culture) and **(B)** in the presence of M. leprae antigen stimulation (M. leprae-stimulated culture). Quantitative analysis was performed by Cytometric Beads Array (CBA) according to manufacturer instructions. The results are expressed in pg/mL and presented in boxplot format, indicating the median values (min-max values and 25^th^ and 75^th^ interquartile range). Comparative analysis between subgroups was carried out by the Mann-Whitney test. Significant differences are indicated by connecting lines and underscored by * (p<0.05) and ** (p<0.01). Color background (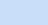 and 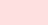, respectively) were used to highlight the chemokines and cytokines with increased levels in HHC(PB) and HHC(MB).

The levels of chemokines (CXCL8, CCL2, CXCL9, CCL5, CXCL10) and cytokines (IL-6, TNF, IFN-γ, IL-17, IL-4, IL-10 and IL-2) were measured in the supernatant collected upon *in vitro* culture of PBMC from HHC(PB) and HHC(MB) and the results shown in the **Figure 1**. Data analysis demonstrated that higher levels of CXCL8 were observed in unstimulated culture from HHC(PB) as compared to HHC(MB) (**Figure 1A**). Additionally, in *M. leprae*-stimulated culture, higher levels of CXCL8, CCL2, CXCL9 and CXCL10 were observed in HHC(PB) as compared to HHC(MB). Conversely, higher levels of pro-inflammatory cytokines (IL-6, TNF, IFN-γ, IL-17) were detected in the supernatant of PBMC cultures from HHC(MB) as compared to HHC(PB) (**Figure 1B**).

### Chemokine and cytokine signatures of *in vitro* cultured PBMCs from household contacts of leprosy patients

**Figure 2:**
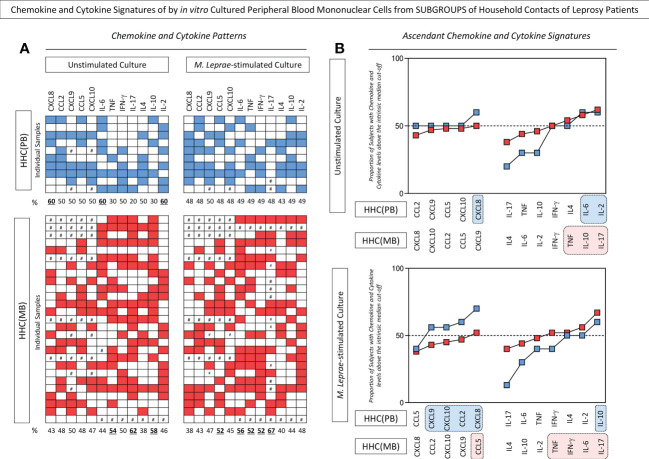
Chemokine and cytokine signatures of *in vitro* cultured peripheral blood mononuclear cells from subgroups of household contacts of leprosy patients. The levels of chemokines (CXCL8, CCL2, CXCL9, CCL5, CXCL10) and cytokines (IL-6, TNF, IFN-γ, IL-17, IL-4, IL-10 and IL-2) were measured in the supernatant collected upon *in vitro* culture of PBMC from household contacts of paucibacillary [HHC(PB) = 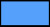, n=10] and multibacillary patients [HHC(MB) = 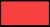, n=27] in the absence of exogenous stimuli (unstimulated culture) and in the presence of M. leprae antigen stimulation (M. leprae-stimulated culture). Quantitative analysis was performed by Cytometric Beads Array (CBA) according to manufacturer instructions. The results are reported as chemokine and cytokine signatures as described in methods. Continuous variables expressed in pg/mL were converted into categorical data using the intrinsic median values as the cut-off for unstimulated culture and M.leprae-stimulated culture as described in methods. The proportion of subjects with chemokine and cytokine levels above the intrinsic median was calculated and data are presented as **(A)** color diagram of individual samples and **(B)** overlayed ascending curves. Color diagrams illustrates the number of subjects with chemokine and cytokine levels above (blue and red squares) or below (white squares) the intrinsic median cut-off, with # indicating missing data. The chemokines and cytokines with proportion of subjects above the 50^th^ percentile were underscored by bold underline format. Overlayed ascending curves were assembled to underscore the chemokines and cytokines with proportion of subjects above the 50^th^ percentile(dashed line) and color rectangle background (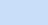 and 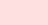, respectively) were used to highlight the exclusive chemokines and cytokines with increased levels in HHC(PB) and HHC(MB).

The levels of chemokines and cytokines measured in the supernatant collected upon *in vitro* culture of PBMC from HHC(PB) and HHC(MB) were further reported as biomarker signatures. The results are shown in **Figure 2**. Color diagrams illustrate the proportion of subjects with levels above intrinsic median cut-off obtained for unstimulated and *M. leprae*-stimulated cultures (**Figure 2A**). Data analysis confirmed the higher levels of CXCL8 and further demonstrated that higher levels of IL-6 and IL-2 observed in unstimulated cultures from HHC(PB). On the other hand, higher levels of TNF, IL-10 and IL-17 were observed in unstimulated cultures from HHC(MB). The chemokine and cytokine signatures of *M. leprae*-stimulated cultures further confirmed that while higher levels of chemokines (CXCL9, CXCL10, CCL2 and CXCL8) along with increased levels of IL-10 were observed for HHC(PB), increased levels of pro-inflammatory cytokines (TNF, IFN-γ, IL-6 and IL-17) besides higher levels of CCL5 were detected for HHC(MB) (**Figure 2B**).

### Association of the *TLR4* rs1927914 polymorphism with the chemokine and cytokine secretion by *in vitro* cultured PBMCs from household contacts of leprosy patients

**Figure 3:**
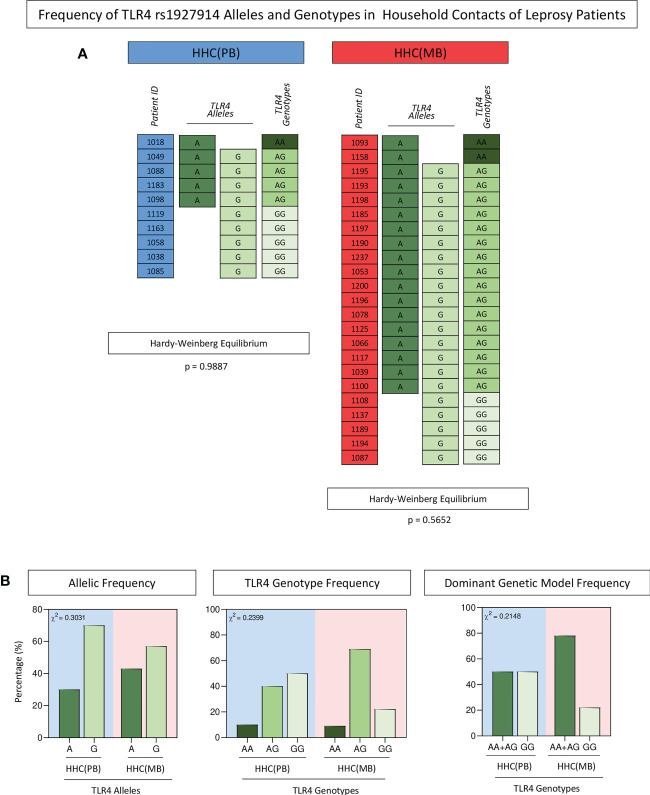
*Frequency of* TLR4 *rs1927914 alleles and genotypes in household contacts of leprosy patients.* The single nucleotide polymorphisms of *TLR4*gene was analyzed as described in methods. **(A)** Color diagrams illustrate the *TLR4* rs1927914 alleles and genotypes profile of household contacts of paucibacillary [HHC(PB) = 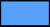, n=10] and multibacillary patients [HHC(MB) = 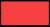, n=27]. **(B)** The allelic (A = 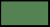 and G = 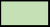), genotypic (AA= 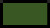, AG =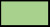 and GG = 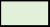) and dominant genetic model (AA+AG = 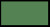 and GG = 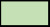) frequencies are shown in bar charts as percentage of subjects within HHC(PB) and HHC(MB) (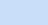 and 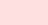 background, respectively). Comparative analysis of *TLR4* rs1927914 alleles and genotypes frequencies between groups was carried out by Chi-square test (χ2) and p values provided in the figure. In all cases, significance was considered at p<0.05.

Overall, the HHC(PB) and HHC(MB) populations displayed HWE. The distribution of allelic (A=40%; G=70% and A=43%; G=57%, respectively) and genotypic frequency (AA=10%; AG=40%; GG=50% and AA=9%; AG=69%; GG=22%, respectively) did not show significant differences between HHC(PB) and HHC(MB). Moreover, the distribution of frequency of carriers of A allele (AA+AG = 50%and 78%, respectively) or the GG major homozygous genotypes (50% and 22%, respectively) did not differ between HHC(PB) and HHC(MB) (**Figure 3**).

**Figure 4:**
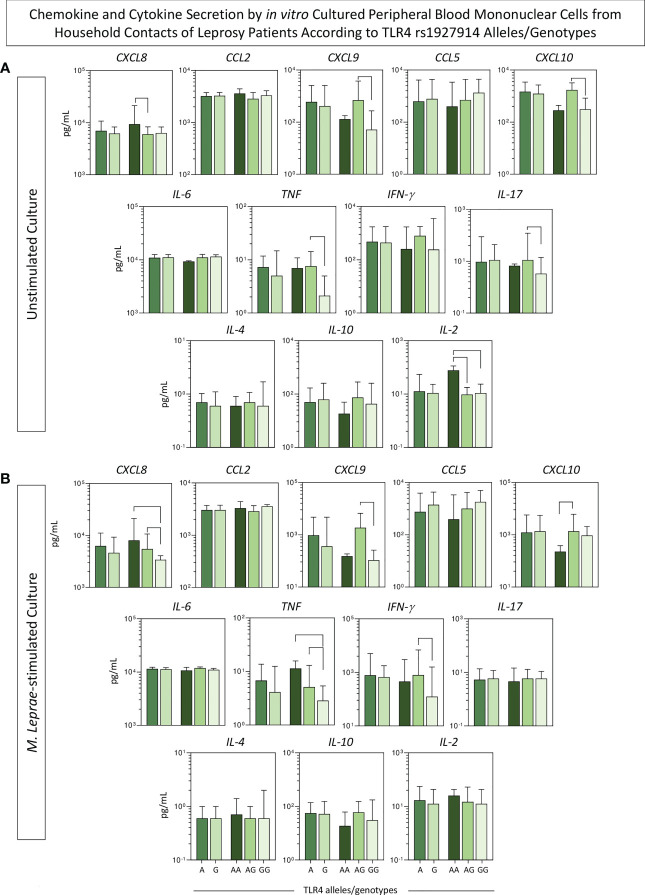
Chemokine and cytokine secretion by *in vitro* cultured peripheral blood mononuclear cells from household contacts of leprosy patients according to TLR4 rs1927914 alleles/genotypes. The levels of chemokines (CXCL8, CCL2, CXCL9, CCL5, CXCL10) and cytokines (IL-6, TNF, IFN-γ, IL-17, IL-4, IL-10 and IL-2) were measured in the supernatant collected upon *in vitro* culture of PBMC from household contacts of leprosy patients categorized according to the TLR4 rs1927914 alleles/genotypes as follows: A (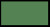, n = 23) and G (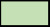, n = 30) alleles as well as AA (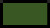, n = 3), AG (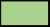, n = 20) and GG (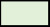, n = 10) genotypes. Data were obtained **(A)** in the absence of exogenous stimuli (unstimulated culture) and **(B)** in the presence of M. leprae antigen stimulation (M. leprae-stimulated culture). Quantitative analysis was performed by Cytometric Beads Array (CBA) according to manufacturer instructions. The results are expressed in pg/mL and presented in bar chats, indicating the median values (25^th^ and 75^th^ interquartile range). Comparative analysis amongst subgroups was carried out by Kruskal-Wallis followed by Dunn’s post-test for multiple comparison between subgroups. Significant differences at p<0.05 are indicated by connecting lines.

Aiming at verifying the association between the SNP *TLR4* rs1927914 and the pattern of immune response, the levels of chemokines and cytokines were measured in the supernatant collected upon *in vitro* culture of PBMC from HHC(PB) and HHC(MB). The groups were then categorized as: carriers of A and G alleles as well as AA, AG and GG genotypes. The results are presented in **Figure 4**. Overall, no significant differences were observed for chemokine and cytokine profiles between A and G alleles. However, higher levels of CXCL8 and IL-2 were observed in unstimulated cultures for the AA genotype. Moreover, in the unstimulated cultures, higher levels of CXCL9, CXCL10, TNF and IL-17 were found for AG genotypes (**Figure 4A**). In general, the GG genotype was associated with lower levels of CXCL8, CXCL9, TNF and IFN-γ in *M. leprae*-stimulated cultures (**Figure 4B**).

### Chemokine and cytokine signatures of *in vitro* cultured PBMCs from household contacts of leprosy patients according to *TLR4* rs1927914 alleles/genotypes

**Figure 5:**
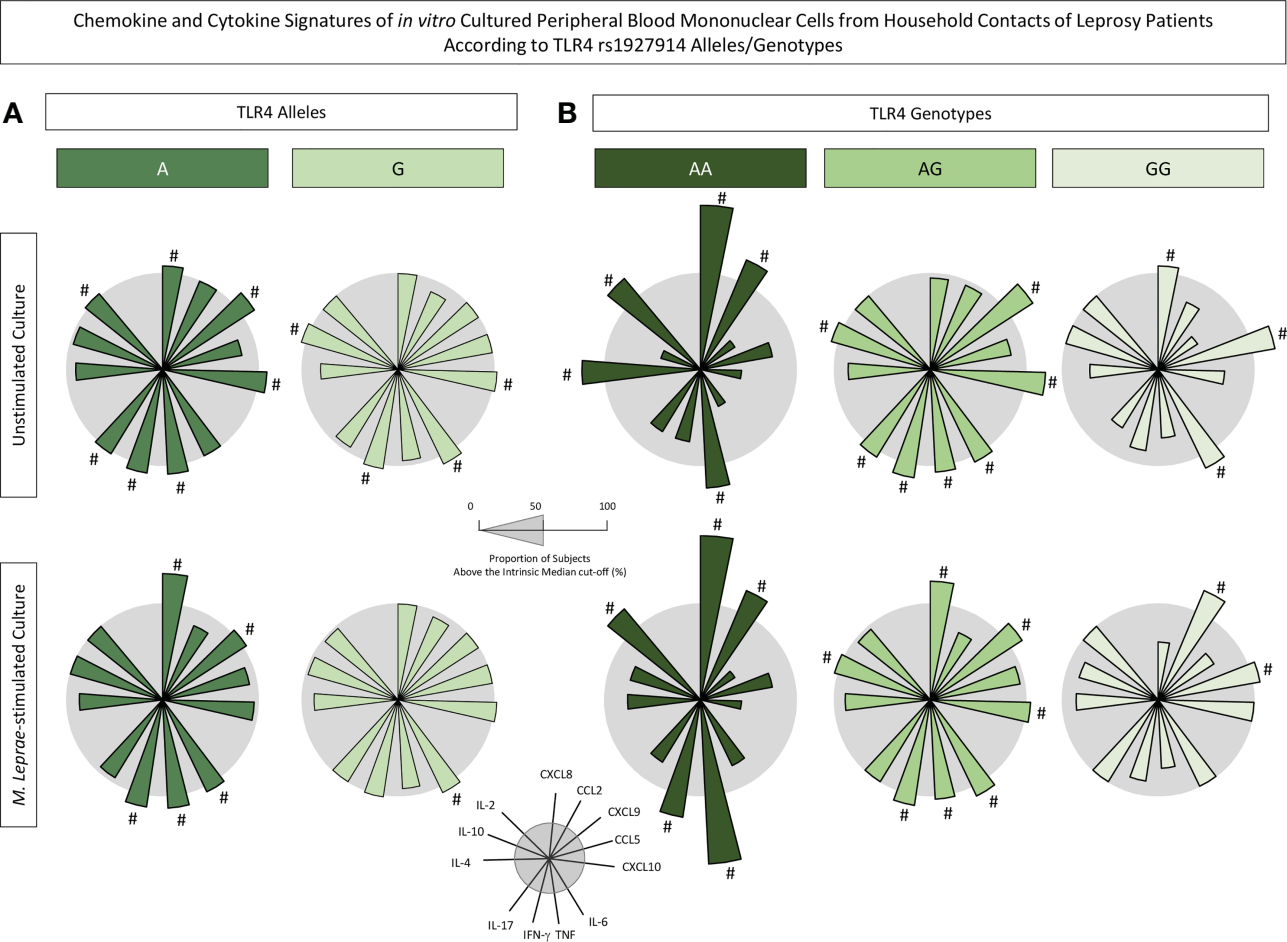
Chemokine and cytokine signatures of *in vitro* cultured peripheral blood mononuclear cells from household contacts of leprosy patients according to TLR4 rs1927914 alleles/genotypes. The levels of chemokines (CXCL8, CCL2, CXCL9, CCL5, CXCL10) and cytokines (IL-6, TNF, IFN-γ, IL-17, IL-4, IL-10 and IL-2) were measured in the supernatant collected upon *in vitro* culture of PBMC from household contacts of leprosy patients categorized according to the TLR4 rs1927914 alleles/genotypes as follows: **(A)** A (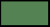, n = 23) and G (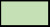, n = 30) alleles as well as **(B)** AA (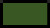, n = 3), AG (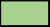, n = 20) and GG (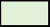, n = 10) genotypes. Quantitative analysis was performed by Cytometric Beads Array (CBA) according to manufacturer instructions. The results are reported as chemokine and cytokine signatures as described in methods. Continuous variables expressed in pg/mL were converted into categorical data using the intrinsic median values as the cut-off for unstimulated culture and M. leprae-stimulated culture as described in methods. The proportion of subjects with chemokine and cytokine levels above the intrinsic median was calculated and data are presented as radar charts with each axis representing each chemokine and cytokine. The chemokines and cytokines with proportion of subjects outside the 50^th^ percentile (gray circle) were underscored by #.

The levels of chemokines and cytokines measured in the supernatant collected upon *in vitro* culture of PBMC from HHC(PB) and HHC(MB) were further reported as biomarker signatures according to the *TLR4* rs1927914 alleles/genotype. The results are shown in **Figure 5**. Radar charts illustrate the proportion of subjects with levels above intrinsic median cut-off obtained for unstimulated and *M. leprae*-stimulated cultures.

Data demonstrated that in unstimulated cultures, the presence of A allele was associated with increased levels of CXCL8, CXCL9, CXCL10, TNF, IFN-γ, IL-17 and IL-2 while the G allele showed an association with higher levels of CXCL10, IL-6, IFN-γ and IL-10 (**Figure 5A**). Notably, upon *M. leprae* antigen recall *in vitro*, it is evident that while the A allele exhibited a more prominent secretion of CXCL8, CXCL9, IL-6, TNF and IFN-γ, the G allele presented only increased levels of IL-6 (**Figure 5A**).

The analysis of chemokines and cytokines according to *TLR4* rs1927914 genotypes further highlighted that AA (CXCL8, CCL2, TNF and IL-2) and AG (CXCL9, CXL10, IL-6, TNF, IFN-γ and IL-10) genotypes were associated with a more prominent secretion of chemokines and cytokines in both culture conditions (**Figure 5B**). These findings further support the clustering of AA and AG genotypes into a dominant genetic model for comparative analysis between AA+AG with GG genotype.

### Chemokine and cytokine secretion by *in vitro* cultured PBMC from subgroups of household contacts of leprosy patients according to the *TLR4* rs1927914 dominant genetic model

**Figure 6:**
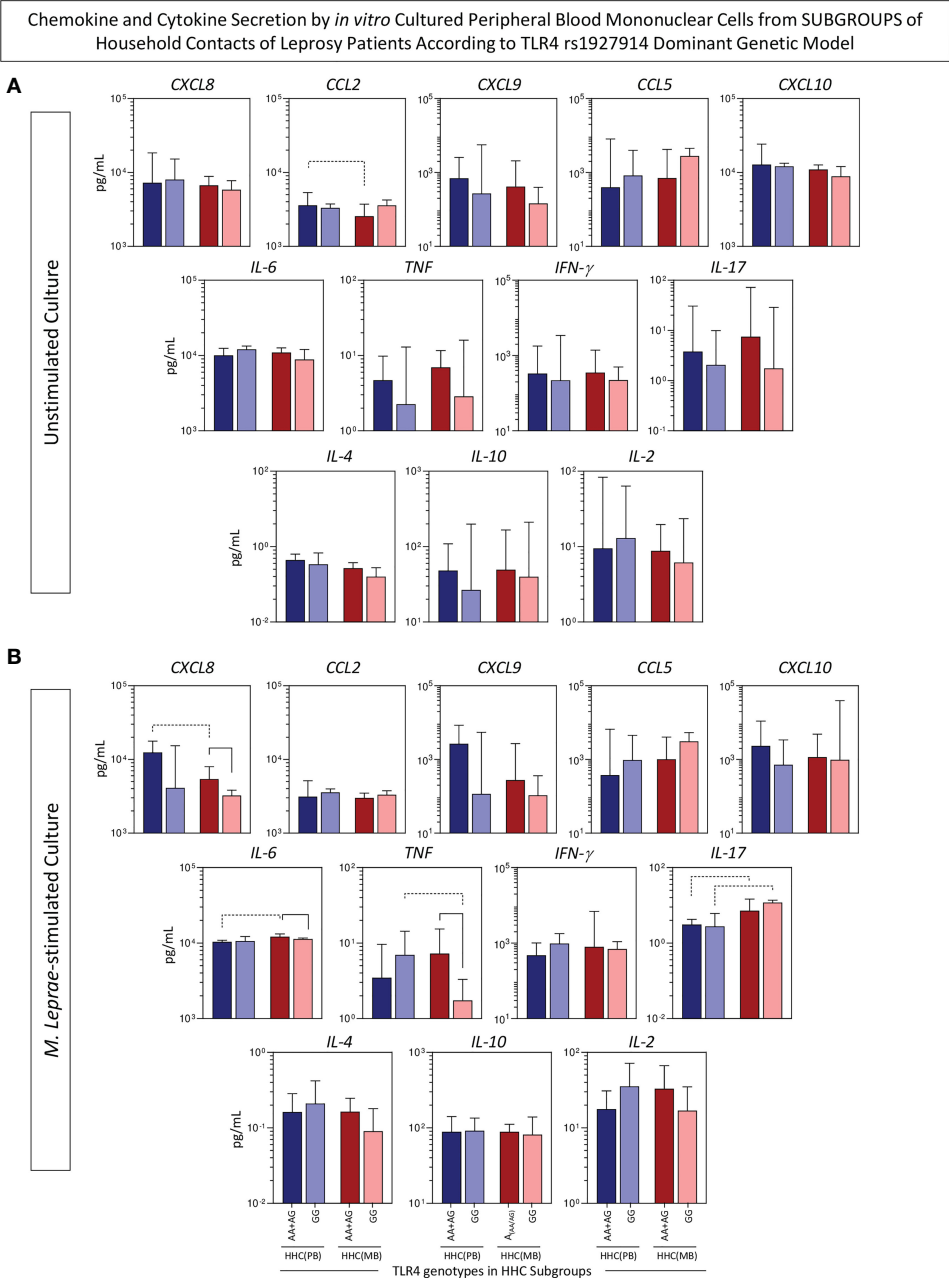
Chemokine and cytokine secretion by *in vitro* cultured peripheral blood mononuclear cells from subgroups of household contacts of leprosy patients according to the TLR4 rs1927914 dominant genetic model. The levels of chemokines (CXCL8, CCL2, CXCL9, CCL5, CXCL10) and cytokines (IL-6, TNF, IFN-γ, IL-17, IL-4, IL-10 and IL-2) were measured in the supernatant collected upon *in vitro* culture of PBMC from household contacts of paucibacillary [HHC(PB), n =10] and multibacilary patients [HHC(MB), n = 23], further categorized according to the TLR4 rs1927914 genotype dominance model, as follows: AA+AG (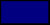, n = 5; 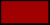, n = 18, respectively) and GG (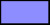, n = 5; 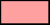, n = 5, respectively).Data were obtained **(A)** in the absence of exogenous stimuli (unstimulated culture) and **(B)** in the presence of M. leprae antigen stimulation (M. leprae-stimulated culture). Quantitative analysis was performed by Cytometric Beads Array (CBA) according to manufacturer instructions. The results are expressed in pg/mL and presented in bar chats, indicating the median values (25^th^ and 75^th^ interquartile range). Comparative analysis amongst subgroups was carried out by Kruskal-Wallis followed by Dunn’s post-test for multiple comparison between subgroups. Significant differences at p<0.05 are indicated by continuous and dashed connecting lines for intragroup and intergroup comparisons, respectively.

An additional strategy was employed to verify the simultaneous impact of SNP *TLR4* rs1927914 concomitant with the operational classification of household contacts. For this purpose, the levels of chemokines and cytokines in the supernatant of *in vitro* culture of PBMC from HHC(PB) and HHC(MB) were compared considering the dominant genetic model (AA+AG *vs* GG). The results are presented in **Figure 6**. Data from unstimulated culture showed that HHC(MB) associated with AA+AG genotypes presented lower levels of CCL2 as compared to HHC(PB) with the same genotype group (**Figure 6A**). Moreover, in *M. leprae*-stimulated cultures, lower CXCL8 and higher IL-6 and IL-17 were observed in HHC(MB) with AA+AG genotypes as compared to HHC(PB) with the same genotype. Additionally, higher TNF and lower IL-17 were observed in HHC(PB) with GG genotype as compared with HHC(MB) with the same genotype (**Figure 6B**). Together, these findings illustrate the impact of differential *M. leprae* exposure over the *TLR4* rs1927914 genotypic background (**Figure 6**).

On the other hand, lower levels of CXCL8, IL-6 and TNF were observed in HHC(MB) with GG genotype as compared to HHC(MB) with AA+AG genotypes, demonstrating that *TLR4* rs1927914 genotypic background may also impact the immune response regardless of the high degree of *M. leprae* exposure (**Figure 6B**).

### Networks of chemokines and cytokines secreted by *in vitro M. leprae-stimulated* PMBCs from household contacts according to the *TLR4* rs1927914 dominant genetic model

**Figure 7:**
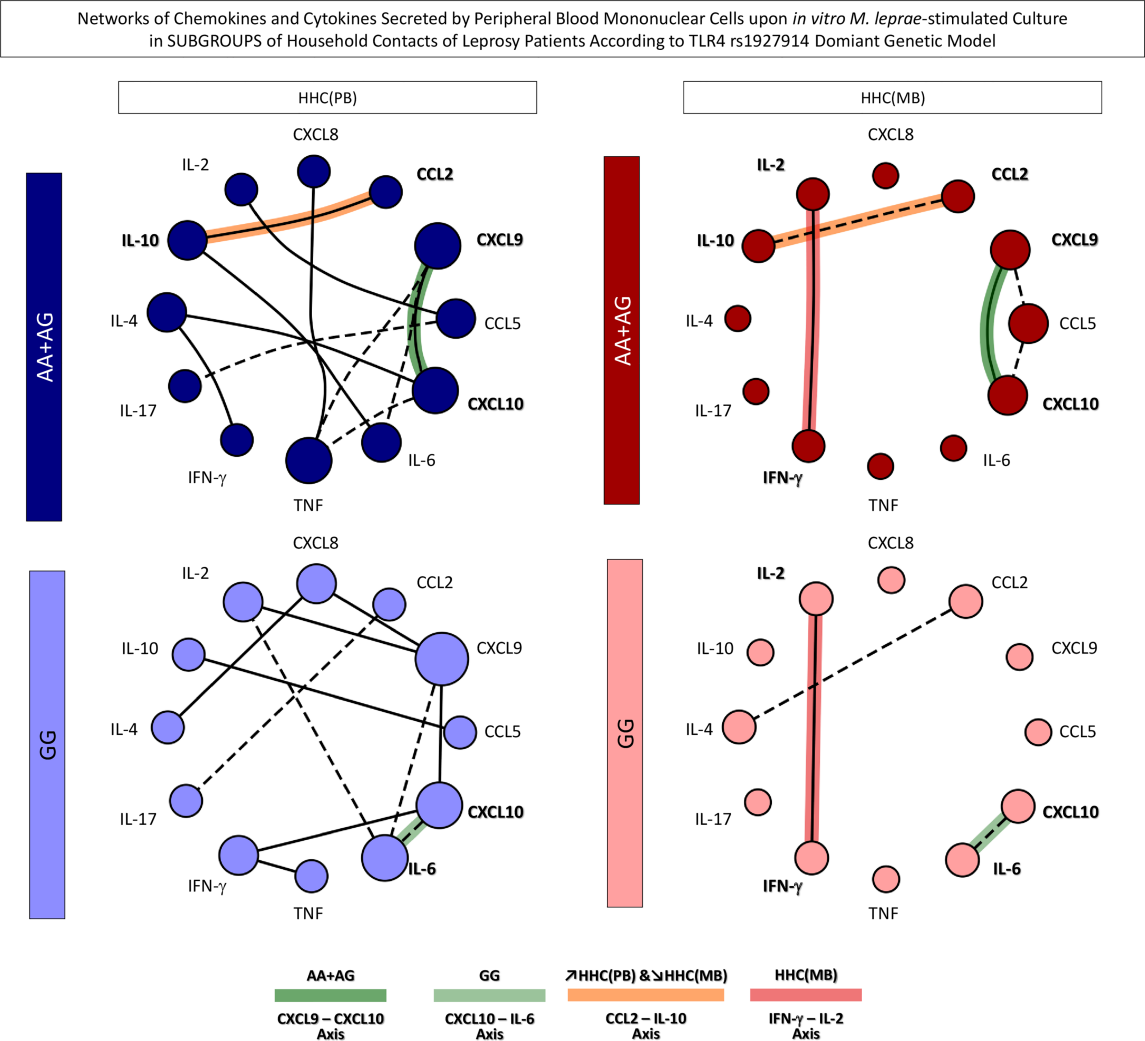
Networks of chemokines and cytokines secreted by *in vitro* M. leprae-stimulated peripheral blood mononuclear cells from subgroups of household contacts according to the TLR4 rs1927914 dominant genetic model. The levels of chemokines (CXCL8, CCL2, CXCL9, CCL5, CXCL10) and cytokines (IL-6, TNF, IFN-γ, IL-17, IL-4, IL-10 and IL-2) were measured in the supernatant collected upon *in vitro* M. leprae-stimulated PBMC from household contacts of paucibacillary [HHC(PB), n =10] and multibacilary patients [HHC(MB), n = 23], further categorized according to the TLR4 rs1927914 genotype dominance model, as follows: AA+AG (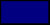, n = 5; 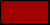, n = 18, respectively) and GG (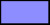, n = 5; 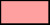, n = 5, respectively). Quantitative analysis was performed by Cytometric Beads Array (CBA) according to manufacturer instructions. The results are presented as circular layout networks with nodes representing each chemokine and cytokine as described in methods. Correlation analysis was employed to construct integrative chemokine and cytokine networks according to significant “r” scores at p<0.05 using Pearson and Spearman correlation tests. Connecting edges illustrate positive (continuous lines) and negative (dashed lines) strong correlations (“r” scores ≥ |0.67|) between pairs of attributes. Color backgrounds were used to underscore selective edges as follows: AA+AG-selective CXCL9 – CXCL10 axis (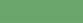), GG-selective CXCL10 – IL-6 axis (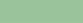), ↗HHC(PB) &↘HHC(MB) CCL2 – IL-10 axis (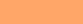) and HHC(MB)-selected IFN-γ – IL-2 axis (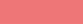).

In order to take an overall snapshot of the simultaneous impact of SNP *TLR4* rs1927914 and the operational classification of household contacts, integrative biomarker networks were assembled considering the “r” scores of significant correlations between chemokines and cytokines. The data are shown in **Figure 7**. Overall, the networks constructs demonstrated that HHC(PB) exhibited more robust connectivity between chemokines and cytokines as compared to HHC(MB). Moreover, it was possible to identify selective edges associated with distinct degrees of *M. leprae* exposure and *TLR4* rs1927914 genotypes. In this sense, *TLR4* rs1927914-associated patterns, referred as AA+GA-selective (CXCL9 – CXCL10) and GG-selective (CXCL10 – IL-6) axis were identified. Conversely, connectivity patterns associated with distinct *M. leprae* exposure profiles were also observed, as demonstrated by the mirrored inverted CCL2 – IL-10 axis displaying positive correlation (↗) in HHC(PB) and negative correlation (↘) in HHC(MB). Besides, an HHC(MB)-selected axis (IFN-γ – IL-2) was also detected (**Figure 7**).

### Performance of chemokines and cytokines secreted *in vitro by M. leprae-stimulated* PBMCs to cluster subgroups of household contacts of leprosy patients

**Figure 8:**
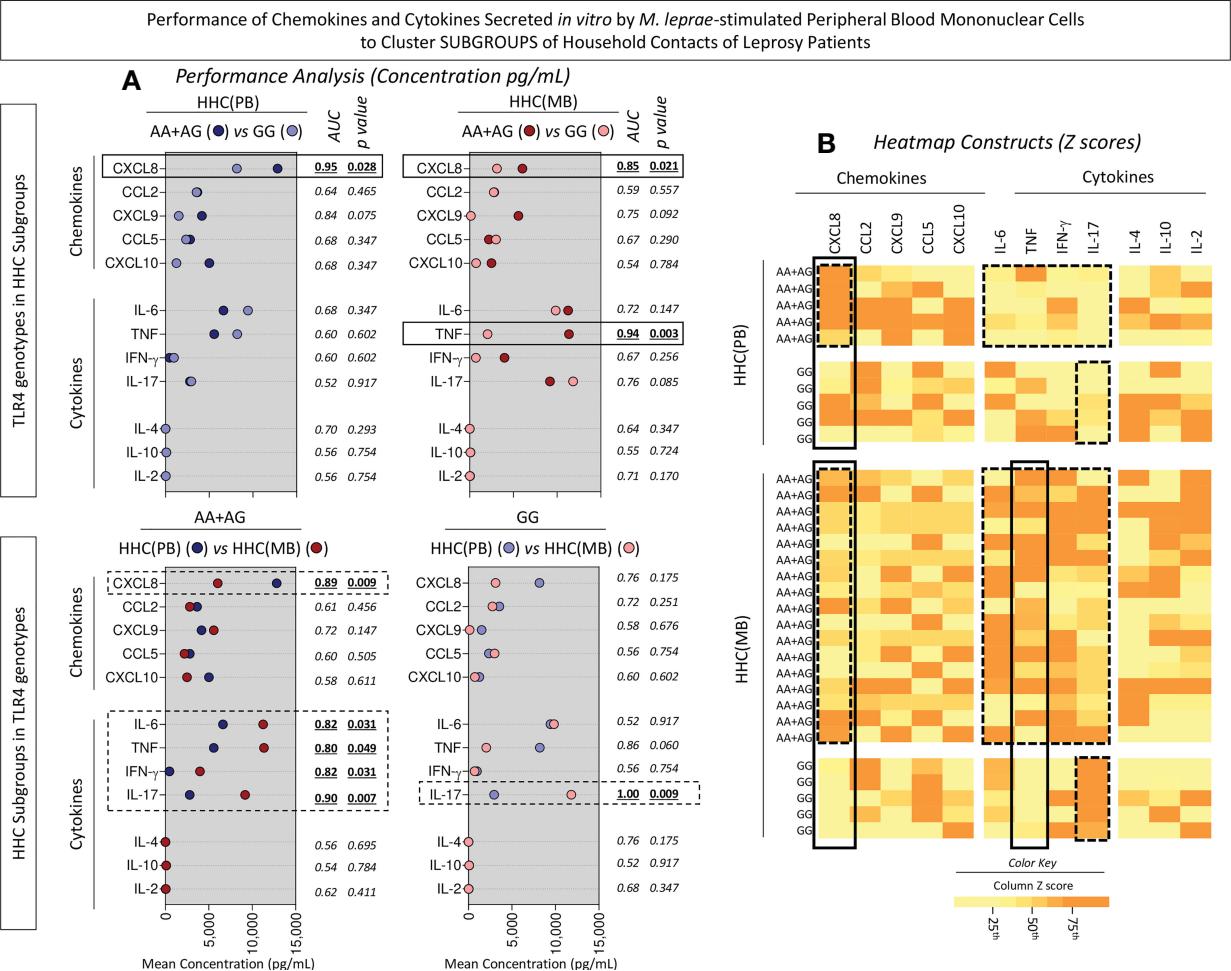
Performance of chemokines and cytokines secreted *in vitro* by M. leprae-stimulated peripheral blood mononuclear cells to cluster subgroups of household contacts of leprosy patients. The levels of chemokines (CXCL8, CCL2, CXCL9, CCL5, CXCL10) and cytokines (IL-6, TNF, IFN-γ, IL-17, IL-4, IL-10 and IL-2) were measured in the supernatant collected upon *in vitro* M. leprae-stimulated PBMC from household contacts of paucibacillary [HHC(PB), n =10] and multibacilary patients [HHC(MB), n = 23], further categorized according to the TLR4 rs1927914 genotype dominance model, as follows: AA+AG (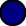, n = 5; 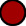, n = 18, respectively) and GG (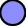, n = 5; 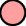, n = 5, respectively). **(A)** Quantitative analysis was performed by Cytometric Beads Array (CBA) according to manufacturer instructions. The results are expressed in pg/mL and presented in scatter plot, indicating the mean values. Comparative analysis between subgroups was carried out by Receiver-operating Characteristic Curves (ROC) and the Area Under the Curve (AUC) used as an indicator of global accuracy. Significant differences at p<0.05 are indicated by bold underline format. Biomarker with higher performance to cluster AA+AG from GG genotypes and HHC(PB) from HHC(MB) are underscored by continuous and dashed rectangles, respectively. **(B)** Heatmap constructs were assembled using conditional formatting in Microsoft Excel Office version 2016. A color key was employed to identify clusters of subjects with distinct (low or high) levels of chemokines and cytokines in each subgroup of household contacts of leprosy patients. The biomarkers able to cluster AA+AG from GG genotypes and HHC(PB) apart from HHC(MB) are highlighted by continuous and dashed rectangles, respectively.

Intending to transpose the laboratorial findings into clinical applied biomarkers, translational analysis was carried out to determine the performance of chemokines and cytokines measured in the supernatant of *in vitro M. leprae*-stimulated PBMC from HHC(PB) and HHC(MB) categorized according to the *TLR4* rs1927914 dominant genetic model. For this purpose, Receiver-operating Characteristic Curves (ROC) were assembled and the Area Under the Curve (AUC) was used as an indicator of global accuracy (**Figure 8**). Data analysis demonstrated that CXCL8 presented outstanding performance to classify both HHC subgroups into AA+AG or GG genotypes with elevated global accuracy (AUC=0.95 and 0.85, respectively). Moreover, TNF also presented elevated performance to classify HHC(MB) into AA+AG and GG genotypes with elevated global accuracy (AUC=0.94) (**Figure 8A**, continuous rectangles).

Several biomarkers presented high performance to classify household contact with AA+AG genotypes according to the *M. leprae* exposure into HHC(PB) and HHC(MB) with elevated global accuracy (AUC for CXCL8 = 0.89, IL-6 = 0.82 TNF=0.80, IFN-γ =0.82 and IL-17 = 0.90). Moreover, IL-17 was able to classify household contact with GG genotypes according to the *M. leprae* exposure into HHC(PB) and HHC(MB) with excellent global accuracy (AUC=1.00) (**Figure 8A**, dashed rectangles).

**Figure 9:**
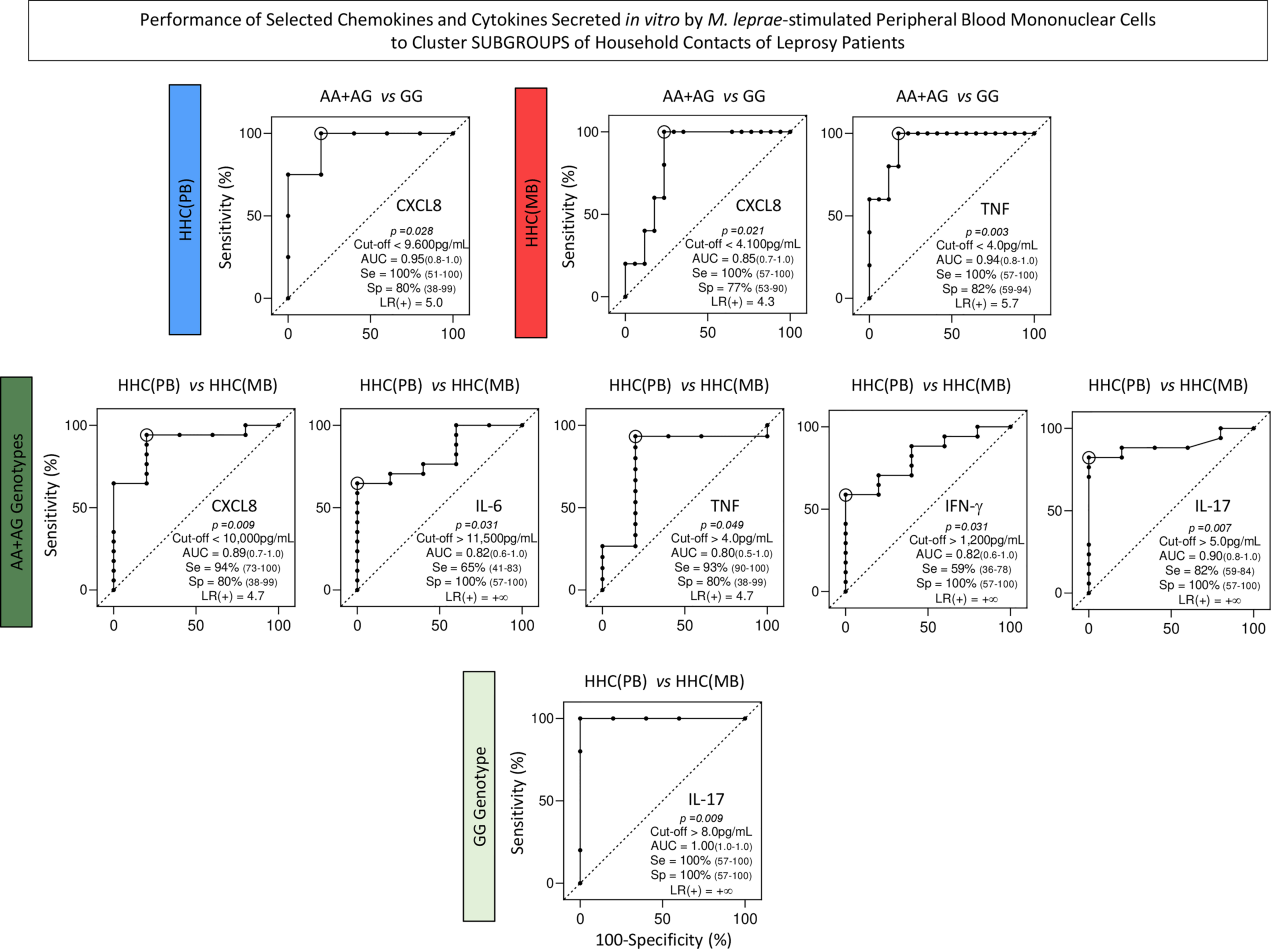
Performance of selected chemokines and cytokines secreted *in vitro* by M. leprae-stimulated peripheral blood mononuclear cells to cluster subgroups of household contacts of leprosy patients. Receiver-operating Characteristic Curves (ROC) were constructed to demonstrate the performance indices for selected chemokines and cytokines (CXCL8, IL-6, TNF, IFN-γ and IL-17) to cluster subgroups of household contacts of paucibacillary [HHC(PB) = 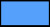] and multibacillary patients [HHC(MB) = 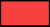] according to the TLR4 rs1927914 dominant genetic model (AA+AG = 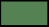 and GG = 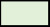). The p values, cut-offs, area under the ROC curve (AUC) with 95% confidence interval (95%CI), sensitivity (Se, 95%CI), specificity (Sp, 95%CI) and likelihood ratio (LR) are provided in the figure. LR = +∞ represents an infinite likelihood ratio.

Heatmap constructs were assembled using the Z score to further illustrate these findings (**Figure 8B**, continuous and dashed rectangles, respectively). ROC curves were constructed to further demonstrate the performance of the selected chemokines and cytokines to cluster subgroups of household contacts according to the *TLR4* rs1927914 dominant genetic model (**Figure 9**).

## Discussion

Leprosy still persists as a relevant public health problem in several countries in Asia, Africa, and Latin America (5). Leprosy classification is usually complex and requires the assessment of several clinical and laboratorial features. Moreover, current attempts are still needed to increase the limited sets of clinical and laboratorial parameters available for predictive diagnosis and monitoring of household contacts of leprosy patients.

The host immune response plays a critical role in the pathophysiology of leprosy and together with genetic factors influences the clinical course of the disease (26). Many immunological biomarkers have been proposed as relevant diagnostic/prognostic tools to classify distinct clinical forms and support prompt treatment onset for effective leprosy control. Immunological parameters have been also suggested as useful laboratories markers to monitor household contacts (HHC)of leprosy patients (27, 28). However, according to Bernard Naafs (29) there is no outstanding laboratorial test to predict whether HHC will develop leprosy after exposure. Since 1973, it has been postulated that higher exposure of HHC to *M. leprae* can impact their immune response and may lead to an increased risk of developing clinical disease (30).

Besides the impact of exposure to *M. leprae* on the immune response of HHC, the host genetic variability can affect the intrinsic susceptibility to leprosy and modulate risk factors to develop clinical manifestations. In this sense, previous studies have shown that single nucleotide polymorphism in TLR genes may contribute to leprosy susceptibility, increasing the risk to develop clinical disease or leprosy reactions (21, 23, 24, 31, 32). While some loci affect intrinsic susceptibility to leprosy (leprosy per se), others modulate risk factors for paucibacillary or multibacillary forms of the disease or for the development of leprosy reactions (31). However, conflicting results are observed amongst genetic studies that may occur due to ethnic diversity as well as differences in the allelic frequencies for TLR genes in indistinct populations.

The main goal of the present investigation was to evaluate the interplay between the operational classification [HHC(PB) and HHC(MB)] and the SNP *TLR4* rs1927914 in household contacts living in Governador Valadares, Minas Gerais State, Brazil, a hyperendemic area for leprosy. The primary endpoint was the impact of these factors on the overall profile of immune response to *M. leprae* antigens. A detailed analysis of chemokines and cytokines secretion was carried out upon *M. leprae*-specific *in vitro* stimulation of PBMCs from subgroups of HHC further classified according to *TLR4* rs1927914 alleles/genotypes.

The results demonstrated that *M. leprae*-stimuli induced higher levels of chemokines (CXCL8, CCL2, CXCL9 and CXCL10) in HHC(PB) as compared to HHC(MB). Conversely, higher levels of pro-inflammatory cytokines (IL-6, TNF, IFN-γ, IL-17) were detected in HHC(MB). The analysis of chemokine and cytokine signatures further corroborate these findings, showing that *M. leprae*-stimuli triggered higher levels of chemokines (CXCL9, CXCL10, CCL2 and CXCL8) in HHC(PB), while increased levels of pro-inflammatory cytokines (TNF, IFN-γ, IL-6 and IL-17) were detected for HHC(MB). The enhancement of pro-inflammatory cytokines in HHC(MB) has been already reported in previous studies from our group (13, 33). The precise relationship between pro-inflammatory cytokines and *M. leprae* exposure is still controversial. Martins et al. (34) have proposed a direct correlation between the levels of IFN-γ and the degree of exposure to *M. leprae*. Moreover, Sampaio et al. (35) have also reported that *M. leprae*-induced IFN-γ production is higher in multibacillary as compared to paucibacillary contacts. Furthermore, the IFN-γ production in response to *M. leprae* antigen stimuli *in vitro* is higher amongst contacts of leprosy patients suggesting a high frequency of sensitization. According to Geluk et al. (36), IFN-γ production induced by *M. leprae* can identify individuals highly exposed to *M. leprae* and therefore at higher risk for developing clinical disease and/or transmitting the bacterium. Conversely, Carvalho et al. (37) demonstrated that the continuous exposure of multibacillary contacts to live *M. leprae* bacilli downregulates the specific cellular immune response against the pathogen (38). It is possible that the extent of response to *M. leprae* antigen primarily depended on the time length of contact and degree of infectiousness of leprosy patients rather than the genetic background dictated by the degree of consanguinity between contacts and the patients (35). Overall, our findings corroborate the hypothesis that as greater the exposure, higher the predisposition to develop the disease (27, 39). Therefore, the prominent pro-inflammatory profile observed in HHC(MB) may be an indicative of early subclinical infection (37).

The analysis of chemokine and cytokine signatures further demonstrated that HHC bearing the A allele of *TLR4* rs1927914presented a prominent secretion of several soluble mediators (CXCL8, CXCL9, IL-6, TNF and IFN-γ). Data analysis according to *TLR4* SNP genotypes further demonstrated AA (CXCL8, CCL2, TNF and IL-2) and AG (CXCL9, CXL10, IL-6, TNF, IFN-γ and IL-10) genotypes were associated with a more prominent secretion of soluble mediators as compared to GG that was associated with lower levels CXCL8, CXCL9, TNF and IFN-γ. These data supported the clustering of AA and AG genotypes into dominant genetic model. Using this dominant genetic model, we have assessed the simultaneous impact of *TLR4* rs1927914 polymorphism and the HHC operational classification on the overall chemokine and cytokine profiles. Data from *M. leprae*-stimulated cultures demonstrated that both: i) differential *M. leprae* exposure and ii) *TLR4* rs1927914 genotypic background have an impact on the immune response of HHC. Lower levels CXCL8 and higher levels of IL-6 and IL-17 were observed in HHC(MB) with AA+AG genotypes. Conversely, higher TNF and lower IL-17 were observed in HHC(PB) with GG genotype.

Integrative biomarker networks were also assembled to evaluate the simultaneous impact of SNP *TLR4* rs1927914. Overall, our findings showed that the resistance-associated genotype (GG) presented an inflammatory axis CXCL10 – IL-6 while the susceptibility-associated dominant genetic model (AA+AG) presented an inflammatory axis CXCL9 – CXCL10. According to Geluk et al. (36), CXCL10 was correlated with exposure to *M. leprae* and, therefore, with the risk of infection. In fact, CXCL10 has been pointed out as a significant biomarker to identify leprosy patients (40–42). It has been shown that increased levels of CXCL10 have been observed in patients able to reduce the bacilloscopic index after multidrug therapy, suggesting that CXCL10 is important to control the bacillary load (43).

Our data demonstrated that lower exposure to *M. leprae* typified by HHC(PB) was associated with an IL-10 modulated axis (↗CCL2 – IL-10), while higher exposure to *M. leprae* exemplified by HHC(MB) did not exhibit a modulatory axis (↘CCL2 – IL-10) and also presented a (IFN-γ –IL-2)-selective axis. It has been shown that CCL2 in association with IFN-γ is a relevant biomarker of subclinical infection of HHC, as also a parameter for early infection monitoring (28).

Intending to transpose the laboratorial findings into clinical applied biomarkers, translational analysis was carried out to determine the performance of chemokines and cytokines to classify HHC according to SNP *TLR4* rs1927914 genotypic background. Our results demonstrated that CXCL8 presented an outstanding performance to classify AA+AG (high levels) from GG (low levels) genotypes in both HHC subgroups. Moreover, amongst AA+AG, CXCL8 was able to cluster HHC(PB) (high levels) from HHC(MB) (low levels). CXCL8 has been pointed out as a potent pro-inflammatory chemokine playing a role in *M. leprae* infection (44). Our findings regarding CXCL8 may appear controversial. Higher levels of CXCL8 were observed in the susceptibility-associated dominant genetic model (AA+AG) as compared to resistance-associated genotype (GG) in both HHC subgroups. However, amongst AA+AG subjects, those with lower exposure to *M. leprae* HHC(PB) presented higher levels of CXCL8. These apparently controversial results, in fact, demonstrated the relevance of carrying out integrated studies of immunological and genetic biomarkers to classify and monitor HHC. Indeed, our results demonstrated that HHC(MB) further classified according to the dominant genetic model (AA+AG) presented lower levels of CXCL8. Ultimately, this finding illustrates the impact of differential *M. leprae* exposure over the *TLR4* rs1927914 genotypic background.

The results showed that TNF has elevated accuracy to classify HHC(MB) into AA+AG (high levels) from GG (low levels) genotype. Previous studies of our group demonstrated that TNF was able to classify HHC according to the operational classification with HHC(MB) presenting low levels as compared to HHC(PB) (16). This apparently contradictory finding further emphasizes the relevance of carrying out immunological studies integrated with genetic analysis to achieve a more accurate classification of HHC.

Our data also demonstrated that IL-17 was able to cluster HHC(MB) (high levels) from HHC(PB) (low levels) regardless of the SNP *TLR4* rs1927914 genotypic background. It is notably the presence of an important IL-17-mediated inflammatory response in the HHC(MB) group regardless of the *TLR4* rs1927914 genotype. The role of IL-17 in leprosy is still controversial. While some studies demonstrate low levels of IL-17 in serum and *in situ*, other reports showed increased levels of IL-17 in leprosy lesions (45, 46). Studies focusing on the IL-17 profile of HHC categorized according to operational classification are still scarce. According to Saini et al. (47), the levels of IL-17 in HHC were high as compared to leprosy patients, suggesting a role of this cytokine as a potential biomarker for early subclinical infection.

Overall, our data support the relevance of investigating the immune response together with genetic profile as potential biomarkers to monitor household contacts. It is important to mention that the present study has some limitations. The small sample size that could reduce the statistical power of our findings requires further investigations with a higher number of subjects. Moreover, the use of multiple comparisons without corrections for confounding variables such as the bacilloscopic index may also impact the conclusions. The study may present a degree of bias, since the participants were selected as a non-probabilistic sampling and therefore, further studies are required to validate these findings prior transposing them as a general statement to a populational basis. Moreover, the observational design must be further validated by longitudinal follow-up of HHC subjects to monitor the development of leprosy disease. Furthermore, simultaneous investigations of household contacts and matching index cases should be considered.

In conclusion, the present study demonstrated that CXCL8 and IL-17 are relevant biomarkers to monitor HHC. While CXCL8 presented a profile closely related to the genetic background, IL-17 appears as an important hallmark of high *M. leprae* exposure. The observation that the degree of *M. leprae* exposure over impacts the TLR genotypic background and vice versa, reinforces that an interplay between these biomarkers should be considered while monitoring HHC. Thus, the integration of immunological and molecular methods to follow-up the development of subclinical infection in HHC is strongly recommended.

## Data availability statement

The raw data supporting the conclusions of this article will be made available by the authors, without undue reservation.

## Ethics statement

The studies involving human participants were reviewed and approved by Ethics Committee from Universidade Federal de Juiz de Fora – UFJF (Research Protocol CAAE #56863016.6.1001.5147). Written informed consent to participate in this study was provided by the participants’ legal guardian/next of kin.

## Author contributions

Study Design: PHFM, JKF and LAOF Funding Acquisition: JKF, ROP, ENS and LAOF. Sample collection, experimental procedures and data acquisition: EHMC, RSG, LBOP, MLS, TNSV and CVR Data Analysis: EHMC, PHFM, JPBS, MLS, CVR, OAMF and LAOF. Writing and reviewing the manuscript: EHMC, PHFM, CVR, OAMF and LAOF. All of the authors read and approved the manuscript.
